# Visual impairment increases the risk of dementia, especially in young males in a 12-year longitudinal follow-up study of a national cohort

**DOI:** 10.1038/s41598-021-91026-4

**Published:** 2021-05-31

**Authors:** Ga-In Lee, Sang Ah Chi, Kyunga Kim, Sang Won Seo, Hee Jin Kim, Tae-Young Chung, Dong Hui Lim

**Affiliations:** 1 Department of Ophthalmology, Samsung Medical Center, Sungkyunkwan University School of Medicine, #81, Irwon-ro, Gangnam-gu, Seoul, 06351 South Korea; 2 Department of Health Sciences and Technology, Samsung Advanced Institute for Health Sciences and Technology, Sungkyunkwan University, Seoul, South Korea; 3 Statistics and Data Center, Research Institute for Future Medicine, Samsung Medical Center, Seoul, South Korea; 4 Department of Digital Health, Samsung Advanced Institute for Health Sciences and Technology, Sungkyunkwan University, Seoul, South Korea; 5 Department of Neurology, Samsung Medical Center, Sungkyunkwan University School of Medicine, Seoul, South Korea

**Keywords:** Dementia, Vision disorders, Risk factors

## Abstract

We investigated the effect of visual impairment (VI) on dementia development in a national cohort. In this 12-year nationwide population-based retrospective cohort study, national data were collected from National Health Insurance Cooperation of South Korea from 2002 to 2017, comprising 799,074 subjects selected from the dementia-free cohort representative of the Korean population. Crude hazard ratios (HRs) as well as age- and sex-adjusted HRs and confidence intervals (CIs) for the development of dementia were estimated using multivariable Cox regression models. VI significantly increased the risk of dementia with a HR of 2.726 (95% CI 2.251–3.300, *p* < 0.0001) after adjusting for age, sex, and interaction between age, sex, and VI. HR of interaction between VI and age for dementia was 0.539 (95% CI 0.436–0.667, *p* < 0.0001). In the sensitivity analysis after adjustment for age, sex, household income level, BMI and other comorbidities, VI showed higher risk for all the type of dementia (*p* < 0.0001). In subgroup analysis of VI, young males showed the highest risk for development of dementia with a HR of 2.687 (95% CI 2.219–3.254, *p* < 0.0001). VI significantly increased the risk of dementia in the study cohort, and young males with VI appeared to be the most susceptible to the development of dementia.

Visual impairment (VI) is well known to be associated with cognitive decline^1–4^. Age-Related Eye Disease Study (AREDS) reported that reduced vision associated with age-related macular degeneration was associated with cognitive impairment in older subjects^4^. The Singapore Malay Eye Study also reported an association between reduced vision with cataract and cognitive dysfunction in an older Asian population^3^. Subjective vision dysfunction based on self-reports was associated with poor cognitive function in a study based in the USA^2^. Furthermore, visual acuity may be an indicator of the neurodegeneration in brain aging^5^.

Likewise, there have been several reports on the associations between VI and dementia^6–8^. Several authors have reported that VI might contribute to a decline in cognition and the development of dementia based on national cohort data^8,9^. Significant associations have been found between visual structures^10–15^ or clinical ophthalmological disorder such as cataract and glaucoma^16–20^ and Alzheimer’s disease (AD). Previous research reported amyloid β, the pathological hallmark of AD, deposits in the crystalline lenses of AD patients^21^. Some studies suggested that retinal pathologies, such as deposits in the macular region, retinal nerve fiber layer thinning^10^, optic disc cupping and retinal microvascular abnormalities^22^ may be related to AD and cognitive impairment^23^. In addition, VI is related to physical and psychological disorders^24^, which themselves are related to the risk of dementia via interaction effects^9,25^.

On the other hands, Hong et al. suggested that the presence of VI was not related with cognitive decline over 5–10 years^26^. Other study reported that open angle glaucoma was not related with increased risk of developing AD^27^. Comprehensively, the relationship between VI and dementia still remains controversial.

Many epidemiological studies in South Korea have been conducted with the nationwide cohorts facilitated by the Korean National Health Insurance Service (NHIS)^24,28,29^. This mandatory universal health insurance system provided the longitudinal database including diagnostic and treatment codes, individual disability information, and socioeconomic data for the entire population of approximately 50 million people. In this study, we used the qualified longitudinal database and constructed a nationwide disease-free cohort to investigate the risk of dementia in VI subjects.

## Patients and methods

### Study population and data collection

For this national cohort study, we used customized health information database from the National Health Insurance Cooperation that can be modified as requested for the purpose of policy and academic research (http://nhiss.nhis.or.kr). The cohort data included personal information, health insurance claim codes (procedures and prescriptions), diagnostic codes from the Korean Standard Classification of Diseases, 7th Revision (KCD-7), which is based on the International Classification of Diseases, 10th Revision (ICD-10)^30^ but with a few changes specific to Korea, death records from the Korean National Statistical Office, medical data and socioeconomic data (residence and income) for each subject over the period from 2002 to 2017.

**Figure 1:**
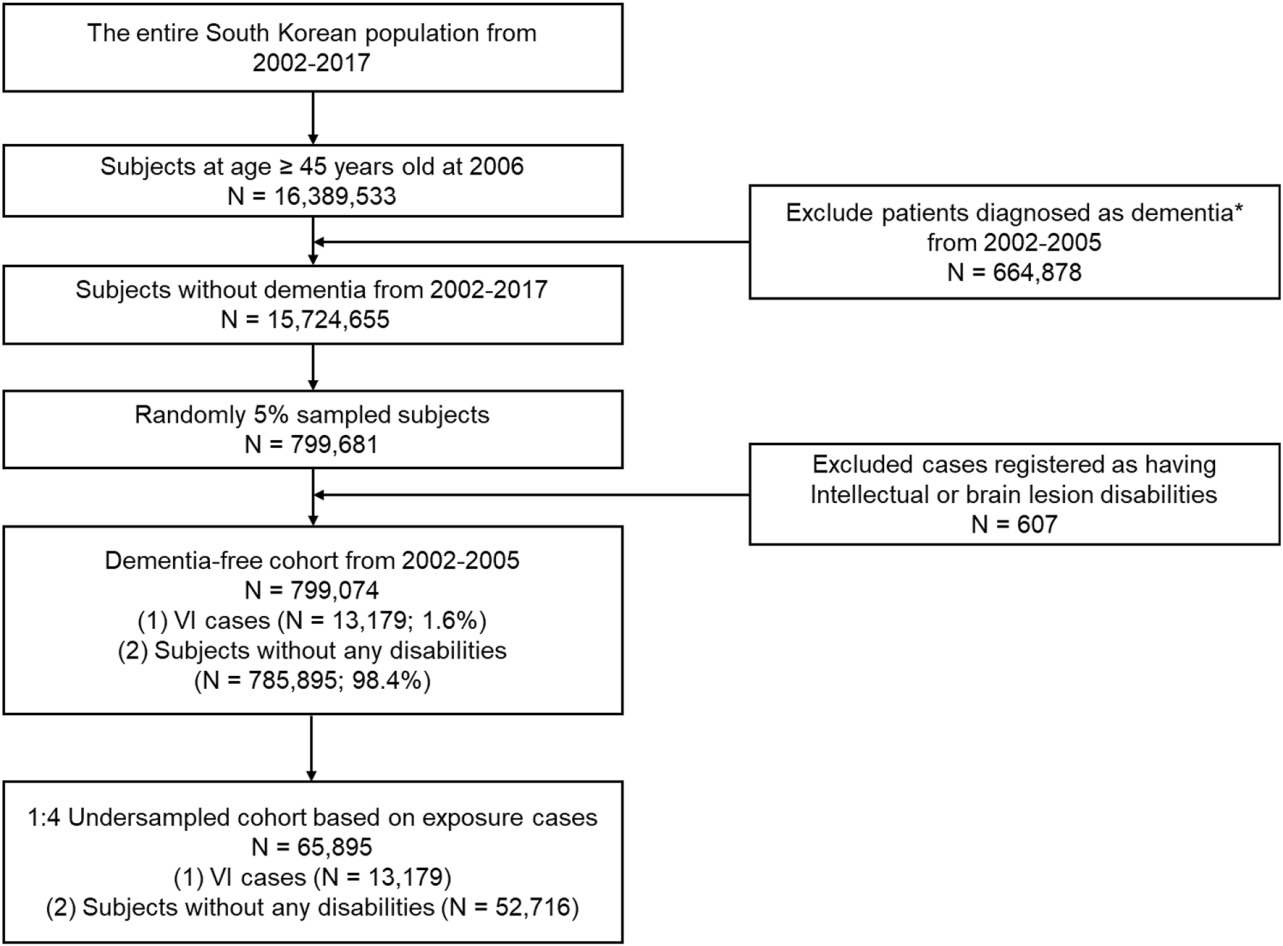
Flow chart of eligible subjects in the dementia-free cohort. VI = visual impairment. *Diagnosis of dementia was based on both International Classification of Diseases-10 diagnosis codes (F00, F01, F02, F03, G30, F05, or G31) and medication prescriptions [acetylcholinesterase inhibitors (donepezil, galantamine, rivastigmine) or NMDA receptor antagonists (memantine)].

We identified a dementia-free cohort representative of the Korean population by washout of dementia cases among individuals aged 45 years and older diagnosed from 2002 to 2005. Diagnosis of dementia was based on both ICD10 diagnosis codes (F00, F01, F02, F03, G30, F05, or G31) and medication prescriptions [acetylcholinesterase inhibitors (donepezil, galantamine, or rivastigmine) or NMDA receptor antagonists (memantine)]. Approximately 5% of subjects were randomly sampled, and subjects with intellectual or brain lesion disabilities that could affect diagnosis of dementia were excluded. Among a total of 799,074 subjects remained in the dementia-free cohort, large differences were observed in the number of participants with VI and without any other legally recognized disabilities: 13,179 subjects had VI without any disabilities (corresponding to only 1.6%), while 785,895 subjects had never been diagnosed with VI or other disabilities (corresponding to 98.4%) from 2006 to 2017. To overcome this imbalance in sample sizes and avoid possible confounding with age and sex, we constructed a reduced dementia-free cohort by age-sex exact matching with a ratio of 1:4. Finally, a total of 65,895 subjects were included in the age- and sex- matched dementia-free cohort, and used in subsequent analyses. Figure 1 shows the processes used to obtain the dementia-free cohort.

This study was approved by the Samsung Medical Center Institutional Review Board (SMC IRB 2018-08-017). The requirement for written informed consent was waived for all subjects by the Samsung Medical Center Institutional Review Board because of the retrospective design and fully anonymised national data. The research was conducted according to the tenets of the Declaration of Helsinki.

### Definitions of exposure and outcomes

Individuals registered as visually impaired persons by the Ministry of Health and Welfare were considered the exposure group. In Korea, legal VI is defined as the presence of any of the following four conditions that show stabilization after at least 6 months of treatment and are not reversed by medication or surgery, with the exception of keratoplasty: 1) best-corrected visual acuity (BCVA) < 20/1000 in the worse eye, 2) BCVA < 20/100 in the better eye, 3) visual field (VF) < 10˚ from the visual axis for both eye, and 4) a binocular VF < 1/2^24^. For the registration of VI status in Korea, an individual must submit a medical certificate issued by an ophthalmologist regarding BCVA, VF and possible reason for VI, and then an assessment committee discusses the feasibility of VI registration^24^. The degree of VI is typically divided into six grades according to the severity of impairment; data are then divided into two grades (severe VI, grades I–II; mild VI, grades III–VI)^24^.

Dementia occurrence was defined by the first prescription of acetylcholinesterase inhibitors (donepezil, galantamine, or rivastigmine) or NMDA receptor antagonists (memantine) and diagnosis with an ICD-10 dementia code (F00, F01, F02, F03, G30, F05, or G31) as described above. We also considered subtypes of dementia: AD (F00, G30), vascular dementia (VaD) (F01) and frontotemporal dementia (FTD) (G31)^31^.

### Other variables

Two age groups were used in the analyses: < 65 years old and ≥ 65 years old. According to body mass index (BMI), individuals were classified as normal (18.5–22.9), underweight (< 18.5), overweight (23–24.9), or obese (≥ 25). We defined household income level based on the ventiles of medical insurance fee as follows: low (1–5 ventiles), middle-low (6–10 ventiles), middle-high (11–15 ventiles), and high (16–20 ventiles). Comorbidities (i.e., depression, dyslipidemia, stroke, coronary heart disease (CHD), hypertension (HTN), and diabetes mellitus (DM)) diagnosed in 2006 using ICD-10 codes were recorded, as these may be associated with an increased risk of dementia.

### Statistical analysis

Chi-square tests or Fisher’s exact tests were used to compare the general characteristics between the exposure and non-exposure groups. Subjects were described as censored if they did not develop dementia until the end of the study or if they were dropped-out from the cohort for some reasons, such as death and insurance disqualification. The cumulative probability of dementia occurrence was estimated by Kaplan–Meier method. Hazard ratios (HRs) for the development of dementia in individuals with versus without VI were assessed via Cox-proportional hazard models in which VI was considered as a time-varying variable to reflect the change of VI status during the follow-up time. Univariable and multivariable analyses were conducted to estimate crude and age-sex adjusted HRs and 95% confidence intervals (CIs), respectively. Subgroup analyses were performed for each age and sex group. The interaction effects between age, sex and VI on dementia were examined. Sensitivity analysis was done to investigate the VI effect with the further adjustment of BMI, household income level, and systemic comorbidities, i.e., depression, dyslipidemia, stroke, CHD, HTN and DM. P-values less than 0.05 were considered statistically significant. All statistical analyses were performed using SAS (version 9.3; SAS Institute, Inc., Cary, NC, U.S.A.) and R Statistical Software (version 3.5; Foundation for Statistical Computing, Vienna, Austria).

## Results

### Baseline characteristics

Among a total of 65,895 subjects in the dementia-free cohort, 13,179 were registered as visually impaired subjects while the remaining 52,716 did not have any disabilities. Dementia occurred in 7607 subjects: 1817 cases (13.8%) among the 13,179 VI subjects and 5790 cases (11%) among the 52,716 subjects without any disabilities.

Of all dementia patients in VI, there were 882 (11.6%) early-onset patients who were diagnosed with dementia when they were younger than 65 years, and 6725 (88.4%) late-onset patients diagnosed when they were 65 years or older. More females (4606; 60.5%) than males (3001; 39.5%) developed dementia. The mean follow-up period and the mean time to dementia diagnosis were 10.28 ± 3.22 years (median: 12.01 years [min, 0.00; max, 12.01]) and 7.30 ± 3.04 years (median: 7.57 years [min, 0.00; max, 12.01]), respectively in the whole cohort. For the subjects with VI, the mean time to diagnosis of dementia was shortened to 5.93 ± 3.26 years (median: 5.96 years [min, 0.01; max, 11.99]).

Table 1 shows the characteristics of the dementia-converter group and the dementia-nonconverter group. There were significant differences in age, female predominance, depression, dyslipidemia, stroke, CHD, HTN, and DM between the dementia and non-dementia groups (*p* < 0.0001).

**Table 1:** Characteristics of study population by presence of dementia (n = 65,895).

Variables, n (%)	Dementia group (n = 7607)	Control group (n = 58,288)	*p* value
**VI**			< 0.0001
No^a^	5790 (76.1%)	46,926 (80.5%)	
Yes	1817 (23.9%)	11,362 (19.5%)	
**Age**			< 0.0001
< 65 years	882 (11.6%)	32,973 (56.6%)	
≥ 65 years	6725 (88.4%)	25,315 (43.4%)	
**Sex**			< 0.0001
Male	3001 (39.5%)	33,489 (57.5%)	
Female	4606 (60.5%)	24,799 (42.5%)	
**VI severity**			
Mild VI	1356 (17.8%)	9269 (15.9%)	< 0.0001
Severe VI	461 (6.1%)	2093 (3.6%)	< 0.0001
**VI with other impairment**			
Hearing impairment	32 (0.4%)	186 (0.3%)	< 0.0001
Kidney impairment	23 (0.3%)	193 (0.3%)	1.0000
**Comorbidity**			
Depression	1060 (13.9%)	4736 (8.1%)	< 0.0001
Dyslipidemia	1924 (25.3%)	12,112 (20.8%)	< 0.0001
Stroke	896 (11.8%)	3087 (5.3%)	< 0.0001
Coronary heart disease	1525 (20%)	8078 (13.9%)	< 0.0001
Hypertension	4314 (56.7%)	22,404 (38.4%)	< 0.0001
Diabetes mellitus	2805 (36.9%)	15,055 (25.8%)	< 0.0001
**BMI**^**b,c**^			< 0.0001
Underweight	311 (4.1%)	1442 (2.5%)	
Normal	2112 (27.8%)	16,558 (28.4%)	
Overweight	1323 (17.4%)	12,862 (22.1%)	
Obese	1583 (20.8%)	16,991 (29.2%)	
**Household income level**^**b,d**^			< 0.0001
Low	1296 (17%)	10,678 (18.3%)	
Middle-low	1093 (14.4%)	10,436 (17.9%)	
Middle-high	1618 (21.3%)	14,186 (24.3%)	
High	2509 (33%)	19,079 (32.7%)	

VI = visual impairment; BMI = body mass index.

^a^Subjects who had never been diagnosed with V1 or other disabilities.

^b^There exist missing values in data: 12,713 (19.3%) and 5000 (7.6%) of the entire population for BMI and household income level, respectively.

^c^BMI was classified as normal (18.5–22.9), underweight (< 18.5), overweight (23–24.9), and obese (≥ 25).

^d^According to medical insurance fee, household income level was classified as low (1–5 ventiles), middle-low (6–10 ventiles), middle-high (11–15 ventiles), and high (16–20 ventiles).

### VI as a risk factor for dementia

**Figure 2:**
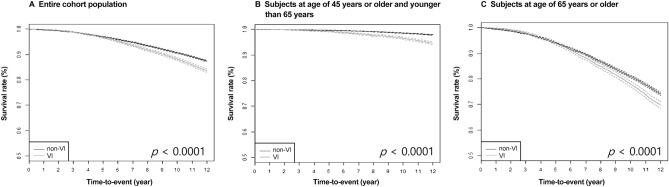
Kaplan–Meier curves for cumulative probability of dementia occurrence according to VI status in (**A**) Entire cohort population, (**B**) Younger age population (< 65 years old), and (**C**) Older age population (≥ 65 years old). Those with VI depicted on the lower line had a higher rate of incident dementia across all age groups by log-rank tests (all *p* < 0.0001).

Kaplan–Meier survival analysis revealed significantly higher cumulative incidence of dementia in the VI group than the non-VI group (log-rank test, *p* < 0.0001) (Fig. 2). This difference in cumulative incidence of dementia was also observed in both age groups (log-rank test, all *p* < 0.0001).

In univariable analyses, VI increased the risk of dementia (HR 1.563, 95% CI 1.485–1.646, *p* < 0.0001). Additionally, age over 65 years, female sex, having depression, dyslipidemia, stroke, CHD, HTN and/or DM, being underweight, and being in the highest household income level increased the risk of dementia (Table e-1).

**Figure 3:**
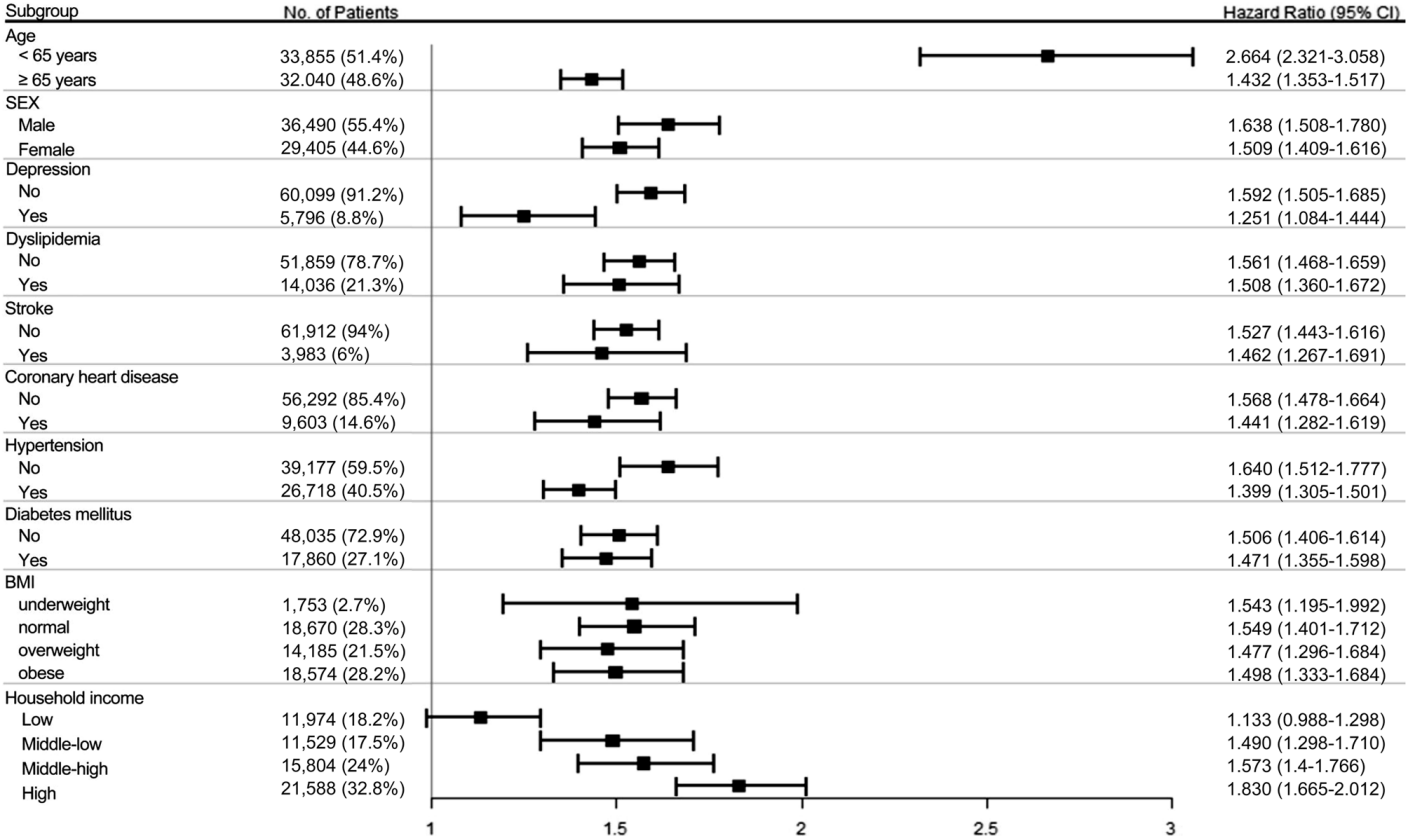
Forest plot for subgroup analyses based on age, sex, systemic comorbidities, BMI and household income level to examine the effect of VI on dementia. Forest plots were generated using the R forest plot package (https://cran.r-project.org/web/packages/forestplot/forestplot.pdf)^57^.

In the multivariable Cox regression analysis with a time-varying effect of VI, we found significantly increased risk of dementia in participants with VI (HR 1.559, 95% CI 1.479–1.644) after adjusting age and sex (Model 1 in Table 2). Individuals 65 years or older and female subjects had a higher risk of developing dementia (HR 10.587, 95% CI 9.867–11.360, and HR 1.522, 95% CI 1.453–1.594, respectively). When the interaction effects of age and sex with VI on dementia were further considered, VI was still a significant risk factor for dementia (HR 2.726, 95% CI 2.251–3.300) (Model 2 in Table 2). While other interactions were not significant, a significant interaction between VI and age (*p* < 0.0001) implied that the effect of VI according to categorized age was significantly different. In subgroup analyses, young males showed the highest risk for development of dementia with a HR of 2.687 (95% CI 2.219–3.254, *p* < 0.0001) compared to old females with a HR of 1.410 (95% CI 1.310–1.517, *p* < 0.0001). We observed that younger subjects with VI at baseline (HR 2.664, 95% CI 2.321–3.058) were more vulnerable to dementia than older subjects with VI (HR 1.432, 95% CI 1.353–1.517) (Fig. 3). The effect of VI did not show difference (*p* for interaction = 0.9219) between males (HR 1.638, 95% CI 1.508–1.780) and females (HR 1.509, 95% CI 1.409–1.616).

**Table 2:** Impact of VI on the risk of dementia via multivariable time-varying Cox regression analyses.

Variables	Model 1^a^		Model 2^b^	
	HR (95% CI)	*p* value	HR (95% CI)	*p* value
VI (yes)	1.559 (1.479–1.644)	< 0.0001	2.726 (2.251–3.300)	< 0.0001
Age group (≥ 65 years)	10.587 (9.867–11.360)	< 0.0001	12.069 (10.674–13.646)	< 0.0001
Sex (female)	1.522 (1.453–1.594)	< 0.0001	1.406 (1.193–1.657)	< 0.0001
VI* age group (yes, ≥ 65 years)			0.539 (0.436–0.667)	< 0.0001
VI* sex (yes, female)			0.986 (0.749–1.300)	0.9219
Age group* sex (≥ 65 years, female)			1.106 (0.929–1.316)	0.2578
VI* age group* sex (yes, ≥ 65 years, female)			0.972 (0.720–1.312)	0.8540

VI = visual impairment; CI = confidence interval; HR = hazard ratio.

^a^Adjusted for age and sex.

^b^Adjusted for age, sex and all interactions between age, sex and VI.

### Sensitivity analysis of the effect of VI on dementia

Subgroup analyses were performed to determine whether VI increased the risk for development of dementia according to household income level, BMI, and the presence or absence of various comorbidities, including depression, dyslipidemia, stroke, CHD, HTN and DM. In all subgroups, VI significantly increased the risk of dementia with HRs ranged from 1.133 to 1.830 (Fig. 3). The effect of VI on dementia development differed among some subgroups: subjects with depression (HR 1.592, 95% CI 1.505–1.685) vs without depression (HR 1.251, 95% CI 1.084–1.444); and subjects with HTN (HR 1.640, 95% CI 1.512–1.777) vs without HTN (HR 1.399, 95% CI 1.305–1.501).

Furthermore, we conducted a multivariable analysis to adjust age, sex, household income level, BMI and comorbid confounders, such as such as depression, dyslipidemia, stroke, CHD, HTN and DM. Even after the adjustment, VI was still a significant risk factor for dementia (HR 2.290, 95% CI 1.946–2.694) (Table 3). These sensitivity analyses suggested that our findings were robust.

**Table 3:** Sensitivity analyses by additional adjustment with comorbidities, BMI and household income level according to sub-types of dementia.

Variables	Any dementia (n = 65,985)	AD (n = 66,640)	VaD (n = 61,890)	FTD (n = 60,340)
	HR (95% CI)	HR (95% CI)	HR (95% CI)	HR (95% CI)
VI (yes)	2.290 (1.946–2.694)	2.261 (1.869–2.735)	3.810 (2.859–5.076)	2.272 (1.352–3.817)
Age group (≥ 65 years)	9.926 (8.995–10.953)	11.616 (10.368–13.013)	11.000 (8.959–13.505)	12.756 (9.321–17.458)
Sex (female)	1.430 (1.352–1.513)	1.503 (1.412–1.600)	1.297 (1.161–1.449)	1.594 (1.343–1.891)
**Comorbidity (yes)**				
Depression	1.405 (1.299–1.521)	1.422 (1.306–1.548)	1.409 (1.208–1.643)	1.226 (0.952–1.578)
Dyslipidemia	0.886 (0.827–0.949)	0.919 (0.853–0.991)	0.856 (0.749–0.978)	0.760 (0.615–0.938)
Stroke	1.517 (1.388–1.658)	1.604 (1.457–1.765)	2.112 (1.806–2.470)	1.519 (1.152–2.002)
Coronary heart disease	1.099 (1.021–1.182)	1.067 (0.985–1.155)	1.081 (0.940–1.244)	1.125 (0.903–1.402)
Hypertension	1.374 (1.290–1.464)	1.315 (1.226–1.409)	1.576 (1.387–1.791)	1.313 (1.085–1.587)
Diabetes mellitus	1.273 (1.198–1.353)	1.236 (1.156–1.322)	1.451 (1.288–1.635)	1.369 (1.140–1.645)
**BMI**^**a,b**^				
Underweight	1.489 (1.316–1.686)	1.566 (1.367–1.794)	1.231 (0.936–1.619)	1.443 (0.972–2.142)
Normal	1.000 (reference)	1.000 (reference)	1.000 (reference)	1.000 (reference)
Overweight	0.797 (0.742–0.856)	0.787 (0.727–0.851)	0.782 (0.680–0.899)	0.844 (0.682–1.044)
Obese	0.676 (0.631–0.724)	0.703 (0.652–0.757)	0.639 (0.558–0.731)	0.662 (0.538–0.814)
**Household income level**^**a,c**^				
Low	1.000 (reference)	1.000 (reference)	1.000 (reference)	1.000 (reference)
Middle-low	0.883 (0.806–0.967)	1.028 (0.928–1.139)	1.027 (0.854–1.235)	0.829 (0.626–1.098)
Middle-high	0.855 (0.788–0.929)	0.977 (0.889–1.074)	1.018 (0.861–1.205)	0.822 (0.638–1.057)
High	0.876 (0.811–0.946)	1.054 (0.966–1.149)	0.995 (0.851–1.165)	0.931 (0.741–1.170)

VI = visual impairment; HR = hazard ratio; CI = confidence interval; AD = Alzheimer’s disease; VaD = vascular dementia; FTD = frontotemporal dementia; BMI = body mass index.

^a^There exist missing values in data: 12,713 (19.3%) and 5000 (7.6%) of the entire population for BMI and household income level, respectively.

^b^BMI was classified as normal (18.5–22.9), underweight (< 18.5), overweight (23–24.9), and obese (≥ 25).

^c^According to medical insurance fee, household income level was classified as low (1–5 ventiles), middle-low (6–10 ventiles), middle-high (11–15 ventiles), and high (16–20 ventiles).

### VI as a risk factor for dementia according to type of dementia

After adjustment for age and sex, VI increased the risk for all types of dementia, namely AD (HR 2.813, 95% CI 2.371–3.339), VaD (HR 4.052, 95% CI 3.109–5.281), and FTD (HR 2.689, 95% CI 1.684–4.294). With further adjustment for household income level, BMI and comorbid confounders, the effect of VI on dementia was still significant for all types of dementia, and greater in VaD (HR 3.810, 95% CI 2.859–5.076) than AD (HR 2.261, 95% CI 1.869–2.735) and FTD (HR 2.272, 95% CI 1.352–3.817) (Table 3).

### Risk of dementia according to the severity of VI and other impairments accompanied

According to the severity of VI, subjects were classified as no VI, mild VI and severe VI. After adjusting for age and sex, we found that severe VI (HR 5.558, 95% CI 4.374–7.062) increased the risk of dementia more than mild VI (HR 2.539, 95% CI 2.167–2.974). We divided the exposure group into three subgroups: VI only, VI with hearing impairment, and VI with kidney impairment. The risk of dementia was greatest in the subjects with VI and kidney impairment (HR 7.501, 95% CI 3.832–14.682), compared to those with VI and hearing impairment group (HR 3.652, CI 1.346–9.907) and those with VI only (HR 2.682, CI 2.307–3.118) (Table e-2).

## Discussion

This nationwide cohort study showed that VI increased the risk of dementia, especially in young males, while dementia was most prevalent among older females. This was consistently observed even after adjustment for age, sex, household income level, BMI and other comorbidities. In subgroup analyses, younger males with VI had the highest risk of dementia, and VaD was the type of dementia most closely associated with VI. Subjects with VI and kidney impairments had a significantly greater risk of dementia than those with VI only or those with VI and hearing impairment. Subjects with severe VI were more at risk of developing dementia than those with mild VI.

Several studies have investigated the association between VI and cognitive function and the impact of VI is still controversial. In the Singapore Malay Eye Study, older people with VI, particularly those with VI due to cataracts, were more likely to have cognitive dysfunction^3^. In a nationally representative sample of the US population, VI based on self-reports was significantly associated with worse cognitive function after adjusting for demographic factors, health, and other possible confounders^2^. In the Fujiwara-kyo eye study, subjects with mild VI had a 2.4-fold higher odds ratio of having cognitive impairment than those without VI after adjusting for age, sex, and length of education^32^. However, in another longitudinal study, no relationship between sensory impairments and decline in cognition has been reported, and only age was found to be significantly associated with possible cognitive decline^26^.

Regarding the effect of VI on the development of dementia, few longitudinal studies were reported^6,9^. Poor vision was reported to be related to the dementia development in the USA; individuals with good vision at baseline had a 63% cut the risk of dementia down over a mean follow-up period of 8.5 years. Moreover, participants with poor vision who did not visit an ophthalmologic clinic had 9.5 times increased the risk of AD^6^. A recent cohort study investigated the relationship between vision loss and 12-year risk of dementia in individuals from three cities in France^9^. This population-based cohort study demonstrated that moderate to severe near VI could be a marker of dementia risk in following years, particularly in participants with depression. Furthermore, self-reported loss of distance visual function was related with an increased risk of dementia. These results are similar with our findings; however, self-reported vision loss possibly differs according to cognitive function capabilities, and no information about BCVA was provided in the previous study. In our study, VI was diagnosed by a certified ophthalmologist, and VI registration was assessed by the government, which would have increased the accuracy and reliability of our findings.

Pathological changes in the visual system, which is composed of the anterior and posterior segments of the eye, optic nerve, and visual cortex, are correlated with dementia. Goldstein et al. reported that Aβ fibrils, which accumulate in the brain of AD patients, are also present in the cytosol of lens fiber cells in individuals with AD, which could promote supranuclear cataract formation^16^. There are several reports of intra-retinal thinning in AD patients based on optical computed tomography (OCT) analysis^12,33,34^. Retinal nerve fiber layer (RNFL) and ganglion cell layer (GCL) thickness are reduced in AD patients compared with healthy controls. A reduction in the number of ganglion cells and in the thickness or the RNFL have also been reported based on histological analysis^35^. In a recent in vivo study, profound tau pathology in the visual system leading to early retinal neuron damage was observed in a mouse model of AD^36^. Studies have also shown degeneration of optic nerve axons in patients with AD^35,37^. As described earlier, senile plaques and neurofibrillary tangles present in the brain lesions of AD patients have also been found in the visual cortex of these patients^38^. The density of these plaques and tangles is greater in area V2, which is associated with visual function, than in V1, particularly in early-onset cases^21,39^. It is possible that VI could be a result of dementia pathology and thus a consequence of dementia rather than a risk factor. There are several reports that VI is associated with AD^7,40,41^. Uhlmann et al. found that the prevalence of VI was higher in the late-onset AD patients than in their age-matched controls, and that the degree of VI was correlated with the severity of cognitive dysfunction^7^. A range of complex visual disturbances were common in AD, which might reflect visuospatial dysfunction related to bi-parietal atrophy, a vulnerable area in AD^42^. Our study showed significantly higher incidence of VI in dementia group, and subjects with severe VI had a 2.19-fold more developing dementia than those with mild VI. This point is expected to be further developed as a remarkable research topic in the future.

Besides, elderly people with VI tend to engage less in cognitively stimulating activities or social activities, and a lack of sufficient sensory input could also lead to neurovascular and neurophysiological changes and thus result in cognitive impairment and development of dementia^8,21,35^. The effect of other sensory impairments on dementia in the elderly have been widely studied^5,43^. Hearing impairment in particular is a proven risk factor for the development of dementia^1,26,43–46^. In early stage hearing impairment, hearing loss increases cognitive load by auditory cortical re-organization in the form of decreased temporal and increased frontal activation^47^. Furthermore, in the elderly, proper listening conditions increase cognitive function and allow the formation of close relationships^48^. These resources may help compensate for the deleterious effects of AD pathology on other cognitive systems in older persons^49^. Hearing loss can also lead to social isolation, which can contribute to dementia^50,51^. VI likely increases the risk of dementia by similar pathomechanisms as detailed above for hearing impairment. In our cohort study, subjects with VI had a significantly increased risk of dementia after adjustment for confounding factors, and VI with hearing impairment had an additive effect on the risk of developing dementia; subjects with VI only had an HR of 2.682 (95% CI 2.307–3.118), while those with VI and hearing impairment had a HR of 3.652 (95% CI 1.346–9.907).

In our study, young males with VI were found to be most vulnerable to developing dementia. Young males had almost twice higher risk of developing dementia than older females. Older individuals and females showed higher risks of dementia development than the general population; however, among VI subjects, a younger age and male sex were factors associated with increased risk for the development of dementia. These results suggest that VI may accelerate the development of dementia among young males. It would contribute to develop or apply different health policies for people with VI to prevent dementia in each age-sex subgroup.

Furthermore, VI with kidney impairment had a stronger additive effect on the risk of developing dementia with a HR of 7.501 (95% CI 3.832–14.682) than VI with hearing impairment or VI without any other impairment. The effect of hearing loss or VI itself on dementia is related with a decline of sensory input, however, that of kidney impairment on dementia is thought to be another mechanism. The fact that kidney changes contribute to the development and rapid progression of dementia supports the strong additive effect of VI with kidney impairment^52–54^. It is also explained that changes in kidney function with increased toxic metabolites lead endothelial dysfunction and loss of vascular integrity^55,56^. Further study to analyze the influence of ischemia on the degree of dementia and VI to explain why VI is more associated with kidney changes than with reduced hearing would be important.

The present study had several limitations. First, there is a gap in time between the occurrence of a disability and registration as a legally disabled person. It may also take some time to receive a diagnosis of VI from the time visual acuity starts to decrease. Furthermore, to receive disability support services in South Korea, there is a waiting period of 6–12 months for registration depending on disability type. This means that a relatively long period of time could elapse from the time that VI develops to being classified as being visually disabled, except in acute events such as trauma. Because VI likely affected cognitive function before the disability was registered, we included all cases where dementia was diagnosed without a latent period after the disability was legally registered by the government into the case cohort group. Second, VI was treated with only for distant vision loss, so we were not able to determine the effect of near vision deterioration on dementia. Lastly, potential dementia cases with subjective memory impairment or amnestic mild cognitive impairment would have been excluded if neurologists did not diagnose dementia or prescribe medications; consequently, we likely underestimated the occurrence of dementia in our cohort.

## Conclusion

In conclusion, individuals with VI exhibited a significantly higher risk for the development of all types of dementia based on the 12-year follow-up period. Notably, the risk was elevated almost twice higher in young males than in old females. VI increased the risk for developing VaD than that for AD. Individuals with severe VI had a higher risk of developing dementia than those with mild VI, and VI with hearing or kidney impairment had an additive effect on the risk of developing dementia. Ophthalmologists and neurologists should be aware that young males with VI are at increased risk for the development of dementia.

## Supplementary Information

Supplementary Table 1.

Supplementary Table 2.

## Data Availability

All datasets generated and/or analyzed during the current study are available from the corresponding author upon reasonable request.
