# Supplementary material for: Visual impairment increases the risk of dementia, especially in young males in a 12-year longitudinal follow-up study of a national cohort
Source: Sci Rep. 2021 May 31;11:11393. doi: 10.1038/s41598-021-91026-4 (PMC8167134; doi:10.1038/s41598-021-91026-4)
Supplement: Supplementary file 1 — Supplementary Table 1. [file 41598_2021_91026_MOESM1_ESM.docx]

Table e-1. Univariate analysis for the risk of dementia during the 12-year follow-up period

| **Variables** | **HR (95% CI)** | ***p* value** |
| --- | --- | --- |
| VI (Yes) | 1.563 (1.485-1.646) | < 0.0001 |
| Age group (≥ 65 years) | 11.133 (10.394-11.924) | < 0.0001 |
| Sex (Female) | 1.850 (1.769-1.934) | < 0.0001 |
| Comorbidity (Yes) |  |  |
| Depression | 1.729 (1.622-1.844) | < 0.0001 |
| Dyslipidemia | 1.298 (1.235-1.364) | < 0.0001 |
| Stroke | 2.662 (2.489-2.846) | < 0.0001 |
| Coronary heart disease | 1.599 (1.514-1.689) | < 0.0001 |
| Hypertension | 2.100 (2.009-2.195) | < 0.0001 |
| Diabetes mellitus | 1.758 (1.680-1.839) | < 0.0001 |
| BMI^a,b^ |  | < 0.0001 |
| Underweight | 1.778 (1.575-2.006) |  |
| Normal | 1.000 (reference) |  |
| Overweight | 0.791 (0.739-0.846) |  |
| Obese | 0.700 (0.657-0.745) |  |
| Household income level^a,c^ |  | < 0.0001 |
| Low | 1.000 (reference) |  |
| Middle-low | 0.890 (0.822-0.963) |  |
| Middle-high | 0.958 (0.891-1.030) |  |
| High | 1.121 (1.050-1.198) |  |

Abbreviations: VI = visual impairment; CHD = coronary heart disease; HTN = hypertension; DM = diabetes mellitus; BMI = body mass index; HR = hazard ratio; CI = confidence interval

^a^ There exist missing values in data: 12,713 (19.3%) and 5,000 (7.6%) of the entire population for BMI and household income level, respectively.

^b^ BMI was classified as normal (18.5-22.9), underweight (< 18.5), overweight (23-24.9), and obese (≥ 25).

^c^ According to medical insurance fee, household income level was classified as low (1-5 ventiles), middle-low (6-10 ventiles), middle-high (11-15 ventiles), and high (16-20 ventiles).
