# Supplementary material for: Visual impairment increases the risk of dementia, especially in young males in a 12-year longitudinal follow-up study of a national cohort
Source: Sci Rep. 2021 May 31;11:11393. doi: 10.1038/s41598-021-91026-4 (PMC8167134; doi:10.1038/s41598-021-91026-4)
Supplement: Supplementary file 2 — Supplementary Table 2. [file 41598_2021_91026_MOESM2_ESM.docx]

Table e-2. Impact of VI severity and comorbidity with other impairments on the risk of dementia after adjustment with age, sex and interactions with severity and comorbidity, respectively.

| **Variables** | **HR (95% CI)** | ***p* value** |
| --- | --- | --- |
| VI severity |  | < 0.0001 |
| No VI ^a^ | 1.000 (reference) |  |
| Mild VI | 2.539 (2.167-2.974) | < 0.0001 |
| Severe VI | 5.558 (4.374-7.062) | < 0.0001 |
| Age group (≥ 65 years) | 13.185 (12.098-14.370) | < 0.0001 |
| Sex (Female) | 1.496 (1.421-1.575) | < 0.0001 |
| VI* Age group |  | < 0.0001 |
| (Mild VI, ≥ 65 years) | 0.520 (0.443-0.611) | < 0.0001 |
| (Severe VI, ≥ 65 years) | 0.363 (0.285-0.463) | < 0.0001 |
| VI* Sex |  | 0.0623 |
| (Mild VI, Female) | 1.022 (0.909-1.149) | 0.7215 |
| (Severe VI, Female) | 0.802 (0.664-0.969) | 0.0222 |
| Comorbidity with other impairments |  | < 0.0001 |
| No VI ^a^ | 1.000 (reference) |  |
| VI only | 2.682 (2.307-3.118) | < 0.0001 |
| VI with hearing impairment | 3.652 (1.346-9.907) | 0.0110 |
| VI with kidney impairment | 7.501 (3.832-14.682) | < 0.0001 |
| Age group (≥ 65 years) | 13.185 (12.098-14.369) | < 0.0001 |
| Sex (Female) | 1.497 (1.422-1.576) | < 0.0001 |
| Comorbidity* Age group |  | < 0.0001 |
| (VI only, ≥ 65 years) | 0.538 (0.462-0.626) | < 0.0001 |
| (VI with hearing impairment, ≥ 65 years) | 0.403 (0.141-1.155) | 0.0907 |
| (VI with kidney impairment, ≥ 65 years) | 0.097 (0.038-0.248) | < 0.0001 |
| Comorbidity* Sex |  | 0.2356 |
| (VI only, Female) | 0.989 (0.887-1.102) | 0.8392 |
| (VI with hearing impairment, Female) | 0.463 (0.214-1.004) | 0.0512 |
| (VI with kidney impairment, Female) | 1.109 (0.485-2.532) | 0.8067 |

Abbreviations: VI = visual impairment; HR = hazard ratio; CI = confidence interval.

^a^ Subjects who had never been diagnosed with V1 or other disabilities.
